# Temporal analysis of mortality from preventable causes in the first 24 hours of life, 2000-2021 *

**DOI:** 10.1590/1518-8345.6696.4080

**Published:** 2023-12-04

**Authors:** Aline Beatriz dos Santos Silva, Luciana Scarlazzari Costa, Paulo Germano de Frias, Ana Catarina de Melo Araújo, Cristine Vieira do Bonfim

**Affiliations:** 1 Universidade Federal de Pernambuco, Recife, PE, Brasil.; 2 Instituto Aggeu Magalhães-Fiocruz, Recife, PE, Brasil.; 3 Becaria de la Coordenação de Aperfeiçoamento de Pessoal de Nível Superior (CAPES), Brasil.; 4 Universidade Estadual de Campinas, Campus Limeira, Limeira, SP, Brasil.; 5 Instituto de Medicina Integral Prof. Fernando Figueira, Recife, PE, Brasil.; 6 Secretaria Estadual de Saúde de Pernambuco, Superintendência de Imunizações e Doenças Imunopreveníveis, Recife, PE, Brasil.; 7 Fundação Joaquim Nabuco, Diretoria de Pesquisas Sociais, Recife, PE, Brasil.

**Keywords:** Cause of Death, Early Neonatal Mortality, Neonatal Nursing, Epidemiological Studies, Forecasting, Public Health, Causas de Muerte, Mortalidad Neonatal Precoz, Enfermeria Neonatal, Estudios Epidemiológicos, Modelos de Predicción, Salud Pública, Causas de Morte, Mortalidade Neonatal Precoce, Enfermagem Neonatal, Estudos Epidemiológicos, Modelos de Predição, Saúde Pública

## Abstract

**Objective:**

to analyze the temporal pattern and estimate mortality rates in the first 24 hours of life and from preventable causes in the state of Pernambuco from 2000 to 2021.

**Method:**

an ecological study, using the quarter as the unit of analysis. The data source was made up of the Mortality Information System and the Live Birth Information System. The time series modeling was conducted according to the Autoregressive Integrated Moving Average Model.

**Results:**

14,462 deaths were recorded in the first 24 hours of life, 11,110 (76.8%) of which being preventable. It is observed from the forecasts that the mortality rate in the first 24 hours of life ranged from 3.3 to 2.4 per 1,000 live births, and the mortality rate from preventable causes ranged from 2.3 to 1.8 per 1,000 live births.

**Conclusion:**

the prediction suggested progress in reducing mortality in the first 24 hours of life in the state and from preventable causes. The ARIMA models presented satisfactory estimates for mortality rates and preventable causes in the first 24 hours of life.

## Abstract (es)

**Objetivo:**

analizar el patrón temporal y estimar las tasas de mortalidad en las primeras 24 horas de vida y por causas evitables en el estado de Pernambuco en el período de 2000 a 2021.

**Método:**

estudio ecológico, teniendo como unidad de análisis el trimestre. La fuente de datos se constituyó por el Sistema de Informaciones sobre Mortalidad y el Sistema de Informaciones sobre Nacidos Vivos. El modelado de series temporales se realizó según el Modelo Autorregresivo Integrado de Promedio Móvil.

**Resultados:**

se registraron 14.462 óbitos en las primeras 24 horas de vida, siendo 11.110 (el 76,8%) evitables. Se observa para los pronósticos ( *forecasts*) que la tasa de mortalidad en las primeras 24 horas de vida registro una variación de 3,3 a 2,4 por 1.000 nacidos vivos, y la tasa de mortalidad por causas evitables de 2,3 a 1,8 por 1.000 nacidos vivos.

**Conclusión:**

la predicción sugirió avances en la reducción de la mortalidad en las primeras 24 horas de vida en el estado y por causas evitables. Los modelos ARIMA presentaron estimaciones satisfactorias para las tasas de mortalidad y por causas evitables en las primeras 24 horas de vida.

## Abstract (pt)

**Objetivo:**

analisar o padrão temporal e estimar as taxas de mortalidade nas primeiras 24 horas de vida e por causas evitáveis no estado de Pernambuco no período de 2000 a 2021.

**Método:**

estudo ecológico, tendo como unidade de análise o trimestre. A fonte de dados foi constituída pelo Sistema de Informações sobre Mortalidade e pelo Sistema de Informações sobre Nascidos Vivos. A modelagem da série temporal foi conduzida segundo o Modelo Autorregressivo Integrado de Médias Móveis.

**Resultados:**

foram registrados 14.462 óbitos nas primeiras 24 horas de vida, sendo 11.110 (76,8%) evitáveis. Observa-se para os *forecasts* que a taxa de mortalidade nas primeiras 24 horas de vida variou de 3,3 a 2,4 por 1.000 nascidos vivos, e a taxa de mortalidade por causas evitáveis variou de 2,3 a 1,8 por 1.000 nascidos vivos.

**Conclusão:**

a previsão sugeriu avanços na redução da mortalidade nas primeiras 24 horas de vida no estado e por causas evitáveis. Os modelos ARIMA apresentaram estimativas satisfatórias para as taxas de mortalidade e por causas evitáveis nas primeiras 24 horas de vida.

**Highlights:**

**(1)** ARIMA is a modeling that is applicable to mortality in the first 24 hours of life.

**(2)** The predictions made show a decrease during the period from 2022 to 2026.

**(3)** Subsidy for nursing in care practices and reducing premature deaths.

## Introduction

Neonatal mortality, which occurs in the first 28 days of life, is an important indicator of the health of a population ^(^ 1 ^)^. The closer it is to the day of birth, the greater the risk of death ^(^ 1 ^)^. The first 24 hours of life correspond to the most vulnerable moment for the newborn, as it requires constant and effective care that reduces the risk of unfavorable outcomes ^(^ 2 ^)^. 

The magnitude of neonatal deaths is measured by calculating the neonatal mortality rate (0 to 27 days), which can be analyzed by these components: early neonatal (0 to 6 days) or late neonatal (7 to 27 days) ^(^ 3 ^)^. Between the years of 1990 and 2019, the global neonatal mortality rate declined from 36.7 to 17.5 per 1,000 live births and in Brazil it went from 25.3 to 7.9 deaths per 1,000 live births ^(^ 4 ^)^. Approximately 75% of deaths in the neonatal period occur in the first week of life, and the first 24 hours of life represent an important proportion (25% to 45%) of global neonatal mortality ^(^ 5 ^)^. 

Among the major Brazilian regions, the mortality rate in the first 24 hours of life varies. In the time series from 2000 to 2019, the Northeast region stands out with the highest rates, which ranged from 6.1 to 3.8 per 1,000 live births, respectively. During this period, the region that presented the highest percentage of rate reduction was the Southeast (45.2%), followed by the South (42.5%) ^(^ 6 ^)^. In the state of Pernambuco, between 2000 and 2016, there were 30,119 neonatal deaths, representing 60.6% of deaths in children under one year of age. Of this total, 68.1% were from preventable causes and occurred in the early neonatal period ^(^ 7 ^)^. 

The relationship between neonatal deaths and healthcare makes these deaths potentially preventable ^(^ 8 ^)^. Methods and classification lists were built to discuss preventable causes of infant and neonatal death ^(^ 9 ^)^. Some methods were developed in different parts of the world, including in Chile (1979), Europe (1980), the United States (1989) and Brazil (2007) such as the Brazilian List of Causes of Deaths Preventable by Interventions from the Unified Health System ( *Sistema Único de Saúde* - SUS in Portuguese) ^(^ 9 ^)^. 

The application of preventability methods makes it possible to identify the main etiological factors involved in neonatal deaths ^(^ 10 ^)^. In Brazil, infant death surveillance is mandatory in health services (public and private) that make up the SUS ^(^ 11 ^)^. This initiative has contributed to the real elucidation of the basic and associated causes and the preventability criteria and to the completeness of the variables in the Death Certificate ( *Declaração de Óbito* - DO in Portuguese) ^(^ 12 ^-^ 13 ^)^. 

Surveillance and monitoring of the temporal behavior of indicators of such early deaths are strategies that support decision-making by policy makers and health managers, in order to improve maternal and neonatal care ^(^ 14 ^)^. 

Analyzing temporal behavior and making predictions regarding infant mortality or its components is a tool with great potential in the field of public health, as it allows knowing the behavior of the phenomenon in question over time, therefore supporting decision making ^(^ 15 ^)^. Different studies recognize the applicability of temporal analysis in understanding infant mortality ^(^ 15 ^-^ 16 ^)^. A study conducted in the state of São Paulo showed a prospect of a drop in infant mortality in the period from 1996 to 2016 ^(^ 16 ^)^. It highlights the potential of temporal analysis stands out through the reliability of forecasting using data available in local healthcare systems ^(^ 16 ^)^. 

Epidemiological studies on mortality in the first 24 hours of life are essential for understanding preventable causes and this way contributing to resolving them. Furthermore, in the national literature there are few studies that use the Autoregressive Integrated Moving Average Model (ARIMA) applied to infant mortality and its components ^(^ 16 ^-^ 19 ^)^. 

Considering that the first 24 hours of life represent a critical period for the survival of the newborn, understanding the evolution over time regarding mortality in this age group is essential to encourage the planning of more appropriate health interventions. Therefore, the objective of this study was to analyze the temporal pattern and estimate mortality rates in the first 24 hours of life and from preventable causes in Pernambuco, from 2000 to 2021.

## Method

### Study design

This is an ecological time series study, in which the quarter constituted the temporal unit of analysis. The choice of the unit of analysis resulted from the minimum assumption of 50 observations that the time series must have to estimate the autocorrelation coefficient, and thus build an acceptable model ^(^ 20 ^)^. The choice of the unit has also taken into account the variability analysis of the rate calculated for the state over a period of one year, in which the quarter showed the lowest variation. 

### Scenario

**Figure 1 -:**
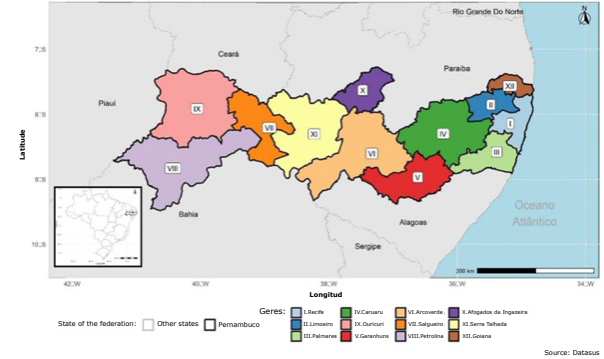
Map of the state of Pernambuco with its division into health regions grouped into macroregions. Pernambuco, Brazil

The study was conducted in the state of Pernambuco (PE), located in the Northeast region of Brazil, with a territorial area of 98,068.021 km², according to the Brazilian Institute of Geography and Statistics (IBGE) ^(^ 21 ^)^. There are 184 municipalities and the state district of Fernando de Noronha, and its capital is the municipality of Recife ^(^ 21 ^)^. The number of live births registered in 2021 in the state was 126,211 ^(^ 6 ^)^. The health territorial organization of the state consists of 12 health regions grouped into four macroregions, namely: *Metropolitana*, *Agreste*, *Sertão* and *Vale do São Francisco/Araripe* ^(^ 20 ^)^ ( Figure 1). 

Primary care coverage in Pernambuco in 2022, according to reports from *e-Gestor Atenção Básica*, ranged from 72.8% to 76.2% from January to December, respectively ^(^ 23 ^)^. Health Region I, which has Recife, the state capital, as its headquarters, concentrates the largest number of obstetricians, as well as the number of intermediate and intensive care beds, with a greater care gap in the mesoregion of the state’s backlands ^(^ 7 ^)^. 

### Population and period

Deaths recorded in the first 24 hours of life and live births from 2000 to 2021 in the state were included.

### Data collection

As a data source, official data from the SUS Information Technology Department (DATASUS) of the Brazilian Ministry of Health were used: Mortality Information System (SIM) and the Live Birth Information System (SINASC) ^(^ 6 ^)^. 

### Data processing and analysis

The calculation of mortality rate consisted of the ratio of the number of deaths in the first 24 hours of life to the total number of live births multiplied by 1,000. The mortality rate due to preventable causes consisted of the ratio of the number of deaths within the first 24 hours of life due to preventable causes to the total number of live births multiplied by 1,000.

The preventability of deaths occurring in the first 24 hours of life was analyzed based on the Brazilian List of Causes of Deaths Preventable by SUS Interventions for children under five years of age, which classifies deaths into three groups of causes: preventable, ill-defined and other causes of death (not clearly preventable) ^(^ 24 ^)^. This classification considers the different technological health densities that are available to the population in the national health context of Brazil ^(^ 24 ^)^. 

Historical series of mortality rates and mortality rates from preventable causes in the first 24 hours of life were analyzed and future values were estimated (prediction). For this analysis, the series considered were quarterly, which implies a frequency of 4. The statistical programming language R version 4.2.2 was used ( https://www.r-project.org/) ^(^ 25 ^)^, using the package *forecast* (version 8.20) for model adjustment and the package *tseries* (version 0.10-53) for applying stationarity and normality diagnostic tests. 

The *forecast* package has the auto.arima function, which applies the variable selection algorithm *stepwise* backwards and forwards to select the best specification for the ARIMA model ^(^ 26 ^)^. In this methodology, several model configurations are tested, and the AIC (Akaike Information Criteria) is measured for each of these configurations ^(^ 27 ^)^. In the end, the model which presented the lowest AIC value is verified; it is chosen as the best configuration among those tested. 

To define the input parameters of the auto.arima function, the Autocorrelation (ACF) and Partial Autocorrelation (PACF) functions were evaluated as well as the Stationarity tests Augmented Dickey-Fuller (ADF), Phillips-Perron (PP) and Kwiatkowski-Phillips-Schmidt-Shin (KPSS) ^(^ 28 ^-^ 30 ^)^. With this evaluation, the input parameters used in the auto.arima function were: testing seasonal series, with a mean different from zero, with *drift* (additional trend term) and non-stationary. Furthermore, the Box-Cox transformation was also used to prevent negative estimates and control the variance of the series. 

The general function of the ARIMA model is a combination of the following parameters: past autoregressive values (p) and noise, moving averages, (q) past, and when the series is not stationary, differentiations (d) are applied to make it stationary ^(^ 20 ^)^. Therefore, the ACF is a function that will help if past values (p) are related to present values, whereas PACF is a function that measures how observations at a given instant of time relate, on average, to observations at previous instants of time, but intermediate observations are known (q) ^(^ 20 ^)^. These concepts can be evolved into seasonal terms. In the ARIMA model, seasonality is given in a multiplicative way ^(^ 31 ^)^. 

Finally, the final model has been validated through Ljung Box’s Q statistical analysis, which tests the hypothesis that the residuals are not autocorrelated, in addition to verifying the normality of the residuals through the Shapiro-Wilk and Jarque-Bera tests ^(^ 31 ^-^ 33 ^)^. After the validation phase, the projection was carried out for the post-sample period of five years (2022-2026), which corresponded to 20 estimated points. Along with the projections, their respective 80% and 95% confidence intervals were made. 

The nomenclature used for the ARIMA models was the classic ARIMA (p,d,q)(P,D,Q)[f] whose indices p, d and q represent, respectively, the autoregressive, differentiation and moving average terms. The terms P, D, Q inform the same previous terms, however, for the seasonal part of the model, [f] indicates the frequency of the series (for this study f=4) and will only appear if the configuration used is seasonal (that is, if there is a term for P, D or Q).

### Ethical aspects

The article was approved by the Research Ethics Committee of the Federal University of Pernambuco under the Certificate of Presentation for Ethical Consideration (CAAE in Portuguese) number 36549020.0.0000.5208.

## Results

**Figure 2 -:**
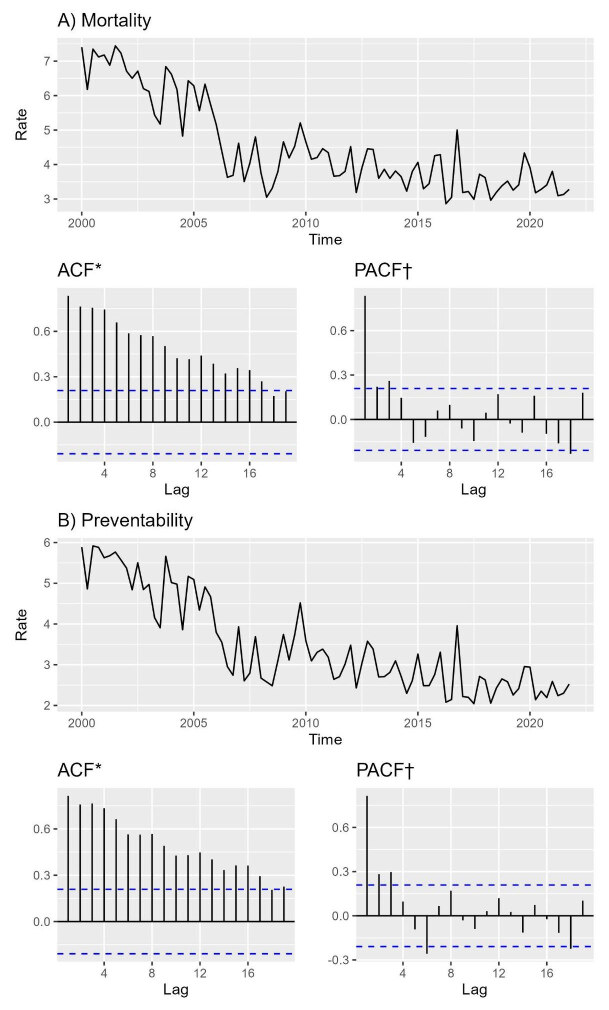
Mortality rates (A) and mortality from preventable causes (B) in the first 24 hours of life and their respective autocorrelation and partial autocorrelation functions. Pernambuco, Brazil, 2000-2021

During the study period, 2000 to 2021, 55,964 infant deaths were recorded, of which 14,462 (25.8%) occurred in the first 24 hours of life. The total number of deaths from preventable causes in the study added up to 11,110 (76.8% of the total deaths recorded in the first 24 hours of life). The mortality rate in the first 24 hours of life in the period varied from 7.8 to 3.2 deaths per thousand live births and the mortality rate in the first 24 hours from preventable causes from 6.6 to 2.5 preventable deaths per thousand live births. The average mortality rate for the period corresponded to 4.6 deaths per 1,000 live births and 3.5 deaths from preventable causes ( Figure 2). 

When evaluating the ACF and PACF of the series ( Figure 2), it is possible to verify a seasonal pattern in the lags of the ACFs/PACFs as well as an indication of a strong trend in the series, due to the slow decay found in the ACFs. The ADF and KPSS tests indicate non-stationarity for both series. The results of all stationarity tests are: mortality with ADF (p-value = 0.51), PP (p-value = 0.01) and KPSS (p-value = 0.01); Preventability with ADF (p-value = 0.57), PP (p-value = 0.01) and KPSS (p-value = 0.01). 

Table 1 presents the results of the various adjusted time series models for the two series. The choice of model was based on the criteria of Akaike ( *Akaike’s Information Criterion* – AIC), which specifies the best (among those tested) ^(^ 18 ^)^. That way, the model chosen for mortality in the first 24 hours was the ARIMA(1,1,1)(2,0,0)[4] and for mortality from preventable causes in the first 24 hours was the ARIMA(1,1,2)(1,0,0)[4] both with a drift term. 

*ACF= Autocorrelation Function; ^†^PACF= Partial Autocorrelation Function

**Table 1 -:** Models adjusted for mortality rates and preventable causes in the first 24 hours of life. Pernambuco, Brazil, 2000-2021

**Mortality rate in the first 24 hours of life**			**Mortality rate in the first 24 hours of life from preventable causes**		
**Model Specifications**	**Has drift** ^‡^	**AIC** ^†^	**Model Specifications**	**Has drift** ^‡^	**AIC** ^†^
ARIMA* ^§^ (1,1,1)(2,0,0)[4]	x	64,108	ARIMA* ^§^ (1,1,2)(1,0,0)[4]	x	20,531
ARIMA* (1,1,1)(2,0,0)[4]		64,841	ARIMA* (1,1,2)(2,0,0)[4]	x	21,259
ARIMA* (1,1,1)(2,0,0)[4]		64,841	ARIMA* (1,1,2)(1,0,0)[4]		21,737
ARIMA* (0,1,2)(2,0,0)[4]	x	64,902	ARIMA* (1,1,2)(0,0,1)[4]	x	22,289
ARIMA* (0,1,2)(1,0,0)[4]	x	64,995	ARIMA* (2,1,2)(1,0,0)[4]	x	22,689
ARIMA* (0,1,1)(2,0,0)[4]	x	65,265	ARIMA* (1,1,3)(1,0,0)[4]	x	22,805
ARIMA* (1,1,1)(1,0,0)[4]	x	65,416	ARIMA* (0,1,1)(2,0,0)[4]	x	22,984
ARIMA* (1,1,2)(2,0,0)[4]	x	65,779	ARIMA* (0,1,1)(1,0,0)[4]	x	23,594
ARIMA* (2,1,2)(2,0,0)[4]	x	66,205	ARIMA* (2,1,3)(1,0,0)[4]	x	24,393
ARIMA* (0,1,2)(0,0,1)[4]	x	66,546	ARIMA* (1,1,1)(2,0,0)[4]	x	24,622
ARIMA* (0,1,2)(0,0,2)[4]	x	66,849	ARIMA* (0,1,1)(0,0,1)[4]	x	24,851
ARIMA* (2,1,0)(2,0,0)[4]	x	67,022	ARIMA* (0,1,2)(2,0,0)[4]	x	24,889
ARIMA* (0,1,3)(2,0,0)[4]	x	67,267	ARIMA* (0,1,2)(1,0,0)[4]	x	25,121
ARIMA* (1,1,1)(0,0,2)[4]	x	67,375	ARIMA* (1,1,2)	x	25,132
ARIMA* (1,1,1)(0,0,1)[4]	x	67,740	ARIMA* (1,1,1)(1,0,0)[4]	x	25,192
ARIMA* (1,1,2)(0,0,2)[4]	x	68,210	ARIMA* (2,1,1)(1,0,0)[4]	x	25,239
ARIMA* (2,1,1)(2,0,0)[4]	x	68,302	ARIMA* (0,1,1)(0,0,2)[4]	x	25,245
ARIMA* (0,1,2)	x	68,487	ARIMA* (0,1,1)	x	26,357
ARIMA* (0,1,1)(0,0,2)[4]	x	68,558	ARIMA* (0,1,3)(1,0,0)[4]	x	26,713
ARIMA* (0,1,1)(0,0,1)[4]	x	68,948	ARIMA* (1,1,0)(2,0,0)[4]	x	33,638
ARIMA* (2,1,1)(0,0,2)[4]	x	69,359	ARIMA* (1,1,0)(1,0,0)[4]	x	41,095
ARIMA* (0,1,1)	x	70,483	ARIMA* (0,1,0)(2,0,0)[4]	x	51,260
ARIMA* (1,1,0)(2,0,0)[4]	x	73,545	ARIMA* (0,1,0)		55,089
ARIMA* (1,1,0)(1,0,0)[4]	x	79,244	ARIMA* (0,1,0)	x	56,924
ARIMA* (1,1,0)(0,0,2)[4]	x	80,429			
ARIMA* (0,1,0)(2,0,0)[4]	x	83,483			
ARIMA* (0,1,0)(0,0,2)[4]	x	87,756			
ARIMA* (0,1,0)		89,744			
ARIMA* (0,1,0)	x	91,465			

*ARIMA= Autoregressive Integrated Moving Average Model; ^†^AIC= Akaike Information Criterion; ^‡^Additional trend term; ^§^Model chosen

**Figure 3 -:**
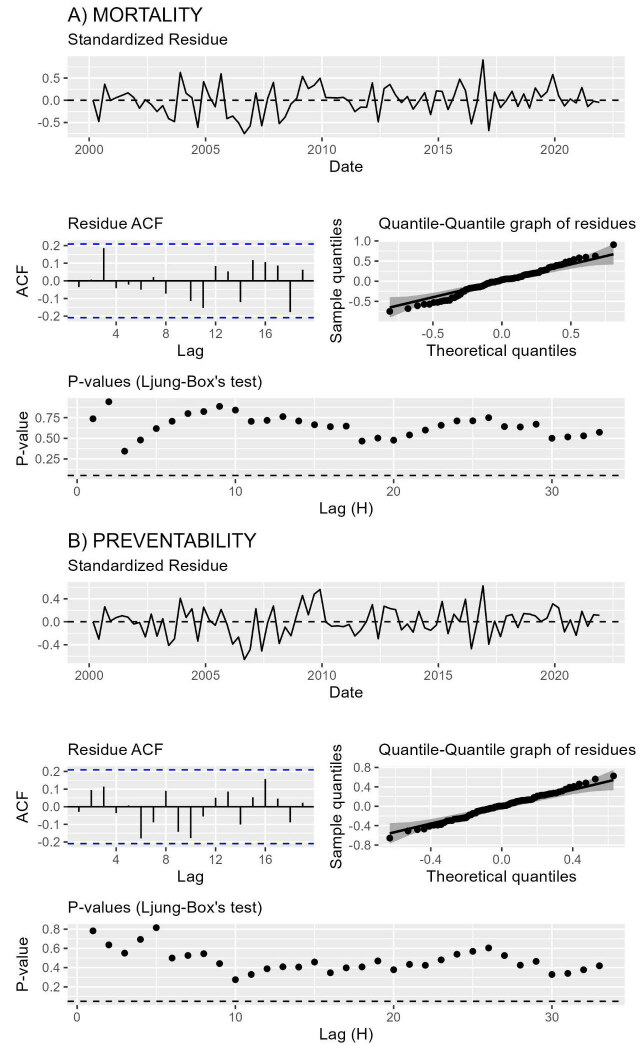
Analysis of residues of the selected models for mortality rates (A) and from preventable causes (B) in the first 24 hours of life. Pernambuco, Brazil, 2000-2021

The Ljung-Box test shows that there is no evidence of association between the residues for any of the series, since the p-values for all lags tested were non-significant ( Figure 3). The ACF graphs for the residuals are in line with the Ljung-Box test. The Shapiro-Wilk and Jarque-Bera tests for mortality and preventability rates were, respectively, 0.45 and 0.98 (mortality rate) and 0.976 and 0.987 (preventability rate), demonstrating that both series presented normality for the residues, in which this evidence can also be verified through the quantile-quantile graph ( Figure 3). Therefore, the models can be considered well adjusted for the rates under analysis. 

*ACF= Autocorrelation Function; ^†^Dashed line equals 5% significance level

**Figure 4 -:**
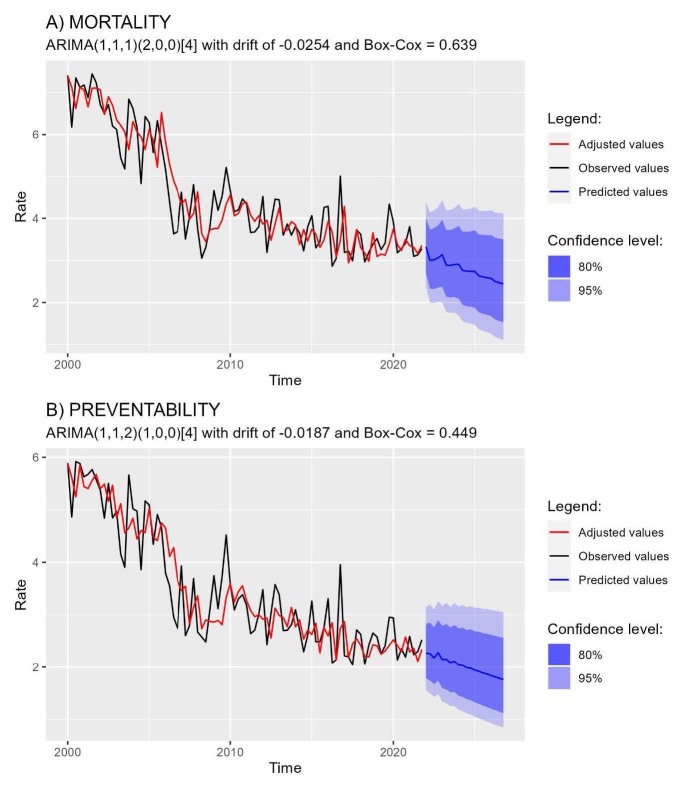
Prediction points (2022 to 2026) of the mortality rate (A) and from preventable causes (B) in the first 24 hours of life. Pernambuco Brazil

The values of the series adjusted by the chosen models presented the same dynamics as the observed values, showing the adjustment of the models ( Figure 4). To carry out the prediction of the two series in question, five years were considered (2022 to 2026), totaling 20 forecast points. It is observed for the forecasts that the mortality rate in the first 24 hours of life ranged from 3.3 to 2.4 per 1,000 live births, and the preventability rate from preventable causes ranged from 2.3 to 1.8 per 1,000 live births. 

*ARIMA= Autoregressive Integrated Moving Average Model

## Discussion

The results of the study show a decreasing trend in the forecast for the years 2022 to 2026. This result shows the importance of forecast studies, to optimize health care and use resources rationally, reducing deaths at such an early age ^(^ 35 ^)^. Early and potentially preventable deaths require universal public interventions and guaranteed care and have a positive impact on reducing mortality ^(^ 7 ^)^. 

Time series analysis studies that aim to estimate health states by predicting indicators is a strategy that should be prioritized, in addition to being a low-cost type of study ^(^ 14 ^-^ 16 ^)^. One of the most common methods for carrying out prediction techniques is the Autoregressive Integrated Moving Average Model (ARIMA), which only requires data arranged on a time basis ^(^ 36 ^)^. 

The results showed an important proportion of preventable deaths relative to the total recorded in the first 24 hours of life. An ecological study that evaluated the temporal behavior of preventable neonatal mortality in large regions of Brazil, from 2000 to 2018, showed that 76% of neonatal deaths could have been avoided ^(^ 35 ^)^. The preventability of deaths occurring in the first 24 hours of life reflects health inequities, which are attributed to socioeconomic, biological and assistance inequalities ^(^ 37 ^)^. 

In Brazil, public policies aimed at the health of women and children were developed in recent decades and improved with the consolidation of the SUS ^(^ 38 ^)^. In particular, with *Bolsa Família*, which transfers income to poor families that comply with conditions related to health and education, and with *Rede Cegonha*, a program which aims to change the model of care for labor and birth, improve access and qualify care practices and management in health care for women and children ^(^ 39 ^-^ 40 ^)^. However, the national scenario from 2016 onwards imposed obstacles in the implementation of initiatives with repercussions on maternal and child health ^(^ 41 ^)^. 

The overall reduction in rates within the study period shows that universal policies, such as coverage of primary health care and, consequently, access to prenatal care with pregnant women identified in a timely manner, were decisive in contributing to this reduction ^(^ 42 ^)^. The seasonality of the series, however, implies that actions need to be intensified, such as early prenatal care, access and resolution of care during childbirth and postpartum ^(^ 42 ^)^. The absence or low investment in socioeconomic improvements and health services aimed at pregnant women and babies are predictors of this type of behavior in the series ^(^ 43 ^-^ 44 ^)^. 

The proportion of preventable deaths through adequate care for women during pregnancy highlighted in the study reinforces the role of habitual and high-risk prenatal care ^(^ 45 ^)^. A previous study in the State of Pernambuco, from 2000 to 2019, showed that the main cause of preventable neonatal death was related to adequate care for women during pregnancy ^(^ 45 ^)^. Another study showed that the *Rede Cegonha* and *Mãe Coruja* programs had an impact on neonatal mortality in Pernambuco. However, in the countryside of the state, where care gaps persist, these programs did not accentuate the downward trend in the neonatal mortality rate due to preventable causes, even with the expansion of prenatal coverage ^(^ 7 ^)^. Therefore, it is recommended to advance qualitatively in the assistance provided to pregnant women and newborns, especially in timely access to prenatal care ^(^ 46 ^)^. 

In contrast to the result found in the present study, in the predicted rates, a study showed that in the neonatal period a slight increase was observed in the predicted rate for a period of five years (2016 to 2020), resulting from some transitions in society, such as: advanced maternal age, obesity/diabetes/hypertension in pregnant women, increased rate of cesarean sections, air pollution, among others ^(^ 47 ^)^. Furthermore, in subsequent years, the question is whether obstacles to the implementation of public policies aimed at women’s and children’s health also do not contribute ^(^ 33 ^)^. 

In line with the predicted decrease in rates described in the results of the present study, a study predicted a consistent decrease of 16% in 2019 and 2020 in the neonatal mortality indicator, going from 33.0 to 17.8 per 1,000 live births, using ARIMA modeling ^(^ 48 ^)^. This implies that the establishment of the Integrated Maternal, Newborn and Child Health Strategy and expansion in the provision of neonatal intensive care assistance are having promising effects in reducing early deaths ^(^ 48 ^-^ 49 ^)^. Using the prediction technique, which is made possible by the method, favors health programming aimed at maternal and child health strategies, by allowing the predicted data to be compared with the goals agreed in local and international health policies ^(^ 9 ^)^. 

As it is an event characterized as stochastic, it must be considered that the findings in the present study are a probability of the event’s behavior. It should be noted, however, that the predictions presented from the technique are influenced by political, social and economic issues, especially when there are scenarios of restrictions on social policies compensating for inequalities in force in society ^(^ 50 ^)^. Public Health Emergencies of National and International Interest deserve special attention, such as that relating to the COVID-19 pandemic, which made the Brazilian population vulnerable and, in particular, pregnant and postpartum women, as well as the healthcare public and maternal and child health service provider network in the country ^(^ 50 ^-^ 51 ^)^. 

The results of the study shown in the diagnostic phase and validation of the models chosen for the two series were relevant, showing that the errors constitute white noise and stating that the adjusted model and the ARIMA specification are adequate. The incorporation of the ARIMA method in the analysis of child deaths, and by components, presents itself as another device for planning interventions in health management ^(^ 9 ^)^. 

As an example of the use of the ARIMA modeling, a study evaluated the performance of some states on infant mortality and showed that the method was satisfactory in its predictions, showing that some states would not be able to reach the 2017 national policy target of 29 deaths per 1,000 live births by 2019 ^(^ 48 ^)^. In this way, forecasting methods can be applied to transform care practices and direct the development of public health policies ^(^ 52 ^)^, in addition to being a methodology that allows the use of data from official sources and low operational costs ^(^ 53 ^)^. 

The use of this type of modeling in the decision-making and health policy-making process in poorer countries is still a challenge, whether due to the absence or fragility of the systematic collection of epidemiological data, or the difficulty of consolidating quality information systems and establishing a culture of data usage ^(^ 53 ^)^. 

It is noteworthy that, due to the period of the study, one should not fail to consider the COVID-19 pandemic, in which the severe acute respiratory syndrome (SARS-CoV-2) culminated in the global health crisis as an emergency starting on the end of December 2019 ^(^ 54 ^)^. Admittedly, public health crises can affect the temporal trend of demographic and mortality indicators ^(^ 55 ^-^ 57 ^)^. A recent study, however, which analyzed trends in fetal and neonatal outcomes during the COVID-19 pandemic, showed that pandemic periods were not associated with a significant change in stillbirth and neonatal mortality rates compared to the baseline period ^(^ 58 ^)^. 

The pandemic has overloaded health services due to the increase in the incidence of serious cases and deaths, causing many essential routine services to become more fragile in ensuring the implementation of essential strategies to combat child deaths - such as the interruption of prenatal care and, consequently, the increase in obstetric complications in emergency services ^(^ 59 ^)^. Therefore, it must be considered that this epidemiological scenario could interfere with the predictions made by the study, which reinforces the relevance of constant monitoring using the ARIMA prediction modeling, providing better information management and assisting in decision making. 

The limitations of the study refer to the use of secondary data, which is subject to underreporting. However, it is worth noting that the SIM has shown improvements in data quality over time ^(^ 7 ^)^. Furthermore, SIM data was used, investigated and qualified by the fetal and infant death surveillance strategy. This strategy, admittedly, contributes to improving notification of the underlying cause and preventability of deaths ^(^ 60 ^)^. Studies conducted in Brazil demonstrated the contribution of this surveillance to defining the underlying cause of death ^(^ 60 ^-^ 61 ^)^. Another limitation concerns the use of the ARIMA modeling, which is based on the premise that the studied event is treated as uniform (linear) behavior during the observed period ^(^ 20 ^)^. It should also be considered that predictions may be affected by the direct or indirect effects of the COVID-19 pandemic on maternal and child health. 

The results of this study can contribute to nursing in identifying the behavior of the mortality rate in the first 24 hours of life, giving visibility to the public health problem in question and offering support for decision making to concentrate resources on care practices to contribute to the reduction of such premature deaths.

## Conclusion

The prediction suggests progress in reducing mortality in the first 24 hours of life in the state and from preventable causes. The ARIMA models presented satisfactory estimates for mortality rates and from preventable causes in the first 24 hours of life.

Despite the reduction observed in mortality in the first 24 hours of life and from preventable causes, there is still a long way to go regarding the determinants of maternal and child health in the state. Efforts are required to qualify and expand the care continuum, which invites the development of additional studies on the labor and birth care network. Therefore, it is expected that the results found can contribute to the formulation of strategies and decision-making with the aim of reducing neonatal deaths.
